# Evolutionary Developments in Interpreting the Gluten-Induced Mucosal Celiac Lesion: An Archimedian Heuristic

**DOI:** 10.3390/nu9030213

**Published:** 2017-02-28

**Authors:** Michael N. Marsh, Calvin J. Heal

**Affiliations:** 1 Luton and Dunstable Hospitals University NHS Trust, and Wolfson College, University of Oxford, Linton Road, Oxford OX2 6UD, UK; 2 Centre for Biostatistics, Faculty of Biology, Academic Health Science Centre, University of Manchester, Manchester M13 9PL, UK; calvin.heal@manchester.ac.uk

**Keywords:** computerised image-analysis, celiac mucosa, Marsh classification, ROC-curve analysis, IEL, lymphocyte immuno-subtypes, mesenteric immune system, invalid Marsh III a,b,c sub-classification

## Abstract

The evolving history of the small intestinal biopsy and its interpretation—and misinterpretations—are described in this paper. Certain interpretative errors in the technical approaches to histological assessment are highlighted—even though we may never be rid of them. For example, mucosal “flattening” does not reduce individual villi to their cores, as still seems to be widely believed. Neither is the mucosa undergoing an atrophic process—since it can recover structurally. Rather, the intestinal mucosa manifests a vast hypertrophic response resulting in the formation of large plateaus formed from partially reduced villi and their amalgamation with the now increased height and width of the inter-villous ridges: this is associated with considerable increases in crypt volumes. Sections through mosaic plateaus gives an erroneous impression of the presence of stunted, flat-topped villi which continues to encourage both the continued use of irrelevant “atrophy” terminologies and a marked failure to perceive what random sections through mosaic plateaus actually look like. While reviewing the extensive 40+ year literature on mucosal analysis, we extracted data on intraepithelial lymphocytes (IEL) counts from 607 biopsies, and applied receiver-operating characteristic (ROC)-curve analysis. From that perspective, it appears that counting IEL/100 enterocyte nuclei in routine haematoxylin and eosin (H&E) sections provides the most useful discriminator of celiac mucosae at histological level, with an effective cut-off of 27 IEL, and offering a very high sensitivity with few false negatives. ROC-curve analysis also revealed the somewhat lesser accuracies of either CD3**^+^** or γδ**^+^** IEL counts. Current official guidelines seem to be somewhat inadequate in clearly defining the spectrum of gluten-induced mucosal pathologies and how they could be optimally interpreted, as well as in promoting the ideal manner for physicians and pathologists to interact in interpreting intestinal mucosae submitted for analysis. Future trends should incorporate 3-D printing and computerised modelling in order to exemplify the subtle micro-anatomical features associated with the crypt-villus interzone. The latter needs precise delineation with use of mRNA in-section assays for brush border enzymes such as alkaline phosphate and esterase. Other additional approaches are needed to facilitate recognition and interpretation of the features of this important inter-zone, such as wells, basins and hypertrophic alterations in the size of inter-villous ridges. The 3-D computerised models could considerably expand our understandings of the microvasculature and its changes—in relation both to crypt hypertrophy, in addition to the partial attrition and subsequent regrowth of villi from the inter-villous ridges during the flattening and recovery processes, respectively.

## 1. Introduction

It may be true that a “Copernican revolution” has seen earlier concepts of celiac disease—perceived as a unitary, gluten-induced disease of the gastro-intestinal tract—changed to one exhibiting multisystem involvements, as well as a growing spectrum now known as gluten-related disorders. These include true gluten sensitivity, gluten allergy, and the more recent “wheat gluten intolerance syndrome”. Nevertheless, aspects of the changes wrought throughout the intestinal tract still remain a central issue for celiac disease diagnosis, as well as for those having a primary interest in the mechanisms bringing about those changes and their structural correlates.

Clinically, our current understandings of ‘celiac disease’ derive from the late 19th century, but as two conditions. Paediatricians used celiac disease following Samuel Gee [1] while adult physicians used ‘idiopathic steatorrhea’. The advent of peroral biopsy techniques led to the realisation (1960–1970) that each constituted a single, lifelong condition [2,3]. These biopsy techniques closely followed Wood’s instrument [4] for retrieving gastric mucosa from patients with achlorhydria. Although Margot Shiner in London pioneered one approach (1956), William H. Crosby’s revolutionary capsule (1958), engineered by Heinz Kugler, enjoyed worldwide usage.

Here, already, we have seen many notable evolutionary advances—Dicke, Wood, Shiner, Crosby—and then Rubin. We therefore suggest that if this collection of papers has its referential foundations in Greek philosophical science, then we should also include Archimedes. That is because many “heureka” moments have been characterised, not as notional views concerning a more generalised pathogenesis, but as sequential moments of inspirational “breakthrough”. These have served in exemplifying—and uniquely advancing—our understandings of each mucosal stage in celiac disease pathogenesis.

It is upon these specific, time-based advances that the evolutionary structuring in our interpretation of mucosal immune-pathology has progressively evolved, and on which this essay is based. At the same time, we note that this review will not deal with the complex issues surrounding enteropathy-associated T-cell lymphoma (EATL): that requires its own detailed account.

## 2. Early (Mis-) Interpretations of Intestinal Biopsies

Science never progresses by step-wise, logically perfect steps. Humankind always prefers comfort of the known against the threatening unknown. Paulley’s operative specimens [5], although rejected in ignorance, were as good as later capsule biopsies. Likewise, Dicke’s new findings about gluten protein in pathogenesis were robustly rejected because of Haas’s “curative” banana diet; his assertions were later vindicated [6,7].

Shiner’s tube gained scant interest, although in those early days when fresh intestinal tissue abnormalities were unknown and awaiting informed interpretation, her classification succeeded. This was (unfortunately) based on her view that mucosal flattening is an ‘*atrophic’* process, probably guided by Wood’s description of true gastric atrophy in pernicious anaemia. Viewed histologically, however, each lesion resembles the other. A closer reading of Wood’s studies would have further indicated that a gross misinterpretation was at stake here. The celiac lesion is not atrophic since on gluten restriction, villous regrowth occurs, as was first shown by Charlotte Anderson, thus becoming another diagnostic yardstick [8]. This misinterpretation persists after more than fifty decades.

Furthermore, more careful correlations between dissecting microscopy and histology would not have extended ‘atrophy’ nomenclature into ‘partial’, ‘subtotal’, and ‘total villous atrophy’. These were histological misinterpretations of mosaic surface plateaus, resulting in reports of ‘branched’, or ‘stunted’, flat-topped ‘villi’ [9]. These were not villi, being far too short (<150 μm, compared with normal 350–600 μm). Again, this second misinterpretation persists today.

Two further novel approaches to mucosal structure came at this time. The first used wax reconstructions leading to the recognition of “basins” and “wells” [10]. Here several individual crypt tubes fed upwards into circular basins, which themselves coalesced into the larger wells ~200 μm in diameter and depth, accommodating up to 20 individual crypt openings. It is regrettable that more extensive use of wax models was not deployed in furthering knowledge.

**Figure 1:**
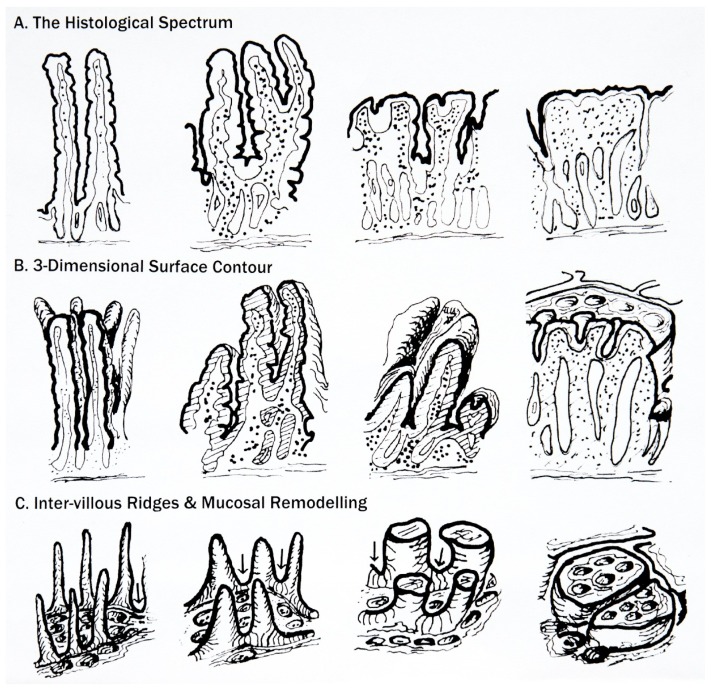
This overview represents intestinal mucosa through its remodelling process from “normal” to typically “flat” celiac appearances [15]. This is not merely an “atrophic” process, but one involving considerable hypertrophic remodelling of the entire mucosal profile. (**A**) The upper series of diagrams, crosswise, illustrate progression as commonly observed in histological section (Marsh Stages 0-III); (**B**) The second line of diagrams depicts the three-dimensional background to flattening, showing the rapid pliancy of villi in their reversion to leaves, ridges, convolutions and finally mosaic plateaus; (**C**) The third line of sketches illustrate *de-epithelialised* mucosae, emphasising the inter-villous ridges (arrowed). Normally, ridges are thin, delicate structures, but as remodelling proceeds, they undergo progressive increments in height and thickness, seemingly filling up the gaps between the now extensively reduced and deformed villi. This fusion results in mosaic plateaus which extend upwards by ~200 μm above the crypt-villus junctional zone (itself complicated by ‘circumvillar basins’ and crypt ‘wells’). At this evolutionary (plateau) stage, it should be appreciated that if a random section passed through consecutive wells, the histologic appearances could well be misinterpreted as “blunted villi”, as often happens in practice. Alternatively, if the sectioning ran between the wells, an entirely flat mucosa would be seen, illustrating one major difficulty inherent in histologic interpretation, especially of the mosaic “terrain”. (Reprinted from Gastroenterology, 151(5), Marsh, Michael N. and Rostami, Kamran, What is a Normal Mucosa? pp. 784–788: Copyright 2016, with permission from Elsevier [15]).

The second approach employed autolysed specimens, thereby exposing the more robust sub-epithelial structures covered by basement membrane [11] including the delicate inter-villous ridges, as also revealed later [12] by scanning EM (see their Figures 1,2,9 and 10). During flattening, theseridges grow higher and thicker, engulfing shortened villi into the characteristic mosaic plateaus [13], whose surfaces lie ~150–200 μm *above* the crypt openings, and confirming histochemical studies [14], in particular of Padykula, who demonstrated the presence of normal (villous) enterocyte enzymes lining their vertical walls (Figure 1). That information is unknown today, and thus contributes little to histological analysis, or its understandings.

## 3. The Immunological Functions of Intestinal Mucosa

Growing disinterest in the idea that celiac enterocytes lack a gluten-digesting “peptidase” (another failure here in recognising the non-specificity of brush border protein digestion) was supplanted by an immune-based pathogenesis. This was buttressed by definitions of the mesenteric immune system by Gowans and Knight who revealed the recirculatory properties of lymphocytes, particularly transference of thoracic duct ‘blasts’ to lamina propria in becoming plasma cells [16]. The latter sustain the local IgA system [17], including its mucosal product—secretory IgA. The functional capacity of this system [18], both throughout the small intestine and the colonic mucosa, was demonstrated [19] elegantly in mice orally primed with the antigen ferritin, an observation ultimately prompting our work in Manchester on rectal gluten challenge [20] and employing logistic regression analysis by Professor Ensari [21]. 

It is important to know that luminal antigen primes naïve lymphocytes in Peyer’s Patches and other primary lymphoid tissues within the intestine to emigrate and recirculate to other mucosal surfaces [22]. This is an important defence against enteric infections, and of protective relevance [23] to lactating humans and animals. Activated recirculating lymphocytes, detected in blood following specific enteric infection in humans, interact with the special β7–MAdCAM-1 receptor exhibited by lamina propria post-capillary venules [24]. More interesting has been the recent demonstration of blood-borne CD4**^+^** gluten-induced T lymphocytes responsive to DQ2-peptide complexes following an oral gluten loading [25,26], again exemplary of the recirculatory potential of mesenteric immune cells reacting to an environmental (dietary) antigen. This has the potential for precise celiac disease diagnosis, and is consistent with the increased numbers of anti-gluten IgA-secreting plasma cells within the mucosa, albeit based on the suspect use [27] of comparative high power fields.

The growing impetus towards diagnostic ‘measurement’ of intestinal biopsies was now based [28] on counts of intra-epithelial lymphocytes (IEL) per 100 villous epithelial cell nuclei. This technique is still the cornerstone of histological diagnosis today, despite its inherent flaw in relating one variable to another variable. “Normal” ranges or diagnostic “cut-off” levels for IEL [28,29,30,31,32,33,34,35,36] (Table 1) range between 20 and 40 IEL, indicating uncertainties over the actual interface. When collated, their fragility becomes strikingly apparent—due to small groups, ill-defined “controls”, interest in other enteropathies (HIV infection), or distribution of IEL at villous tips. Overall, our notions of “the normal range” are distinctly precarious, while the marked overlap between diseasecontrols and celiac patients has never been clarified with additional statistical analyses.

## 4. Re-Evaluating Intraepithelial Lymphocyte (IEL) Counts Derived from the Existing Literature

In order to highlight this impasse, we have reworked and extended previously published data culled from a vast literature (from single case reports to smaller group studies over a 40-year period, as reviewed in this paper) in order to address this issue. In total, data relating to 607 biopsies (386 celiacs) were available for re-evaluation thus providing an important, yet hitherto unknown, extension to the existing literature.

**Figure 2:**
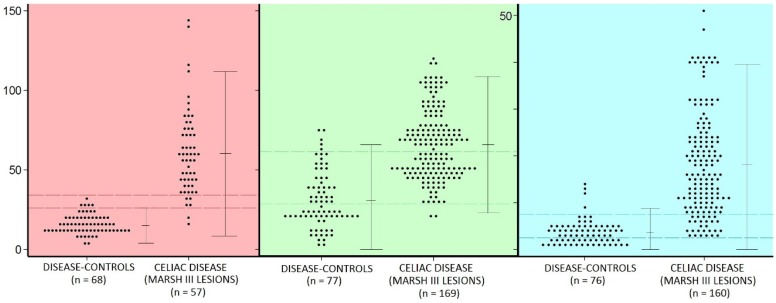
A cumulative assembly of sporadically published reports provided 607 biopsies (386 celiacs), illustrating the numerical distributions of IEL in hematoxylin and eosin (H&E) sections (red), CD3^+^ (green), and γδ^+^ (blue) immuno-subtypes,with their accompanying disease-control groups.(**a**) For IEL (H and E) the mean (±95% Confidence Limits) was 15 (4–26) for controls and 60 (9–111) for celiacs; (**b**) The results for CD3^+^ cells were 31 (0–66) for disease controls, and 66 (23–109) for celiacs; (**c**) For γδ^+^ cells, the mean was 23 (0–39) in the celiac group compared with 4 (0–9) for the controls. Marked overlaps between disease controls and celiac patients (indicated by the paired horizontal lines) occurred with all IEL counts: 43 for histological (H&E) counts, 110 for CD3^+^ counts, and 69 for γδ^+^ cells, respectively.

(a) It is crucial that the considerable overlap between counts of IEL obtained either histologically, or estimated through their immunophenotypes (Figure 2), is acknowledged. From this, two important conclusions follow: (i) that immuno-subtyping IEL does not offer much in the way of improving diagnostic accuracy—again because of massive overlapping; and (ii) that a “normal” IEL count [15] does not exist. 

Each set of counts was not normally distributed. But the ‘normalised’ means after log-transformation differed from the numerical means by only ~5 lymphocytes, indicating that for most practical purposes, IEL counts do not require this treatment. 

**Figure 3:**
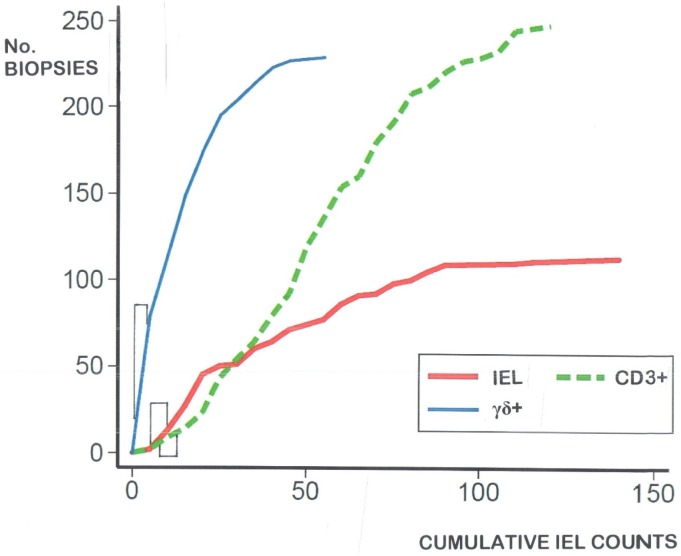
Cumulative IEL counts biopsy-on-biopsy, for histologically counted IEL (red), and immunostained CD3**^+^** (green), and γδ**^+^** (blue) cells. The cumulative overlap between disease control and celiac biopsies is indicated by paired, vertical lines for each of the three data strands.

(b) If these data from each type of measurement (histologically, or by CD3^+^ and γδ^+^ immunophenotyping) are graphically depicted, using a cumulative, biopsy-on-biopsy approach, they all exhibit a continuous, rather than a bi-modal, dose-response (Figure 3). In other words, both control and celiac IEL follow a continuous form of response to gluten ingestion, depending on intrinsic and extrinsic factors: they do not behave as separate clonal populations.

Each graph is reminiscent of a dose-response, consistent with the view that changes in the IEL population, by whichever technique identified, represent graded responses to environmental antigenic challenge. Thus, they do not reveal bimodal behaviour in demonstrating differences between IEL in ‘control’ mucosae, compared with ‘celiac’ mucosae. This explains why there is an overlap and hence no specific, diagnostic cut-off for any of the lymphocyte populations illustrated. 

**Figure 4:**
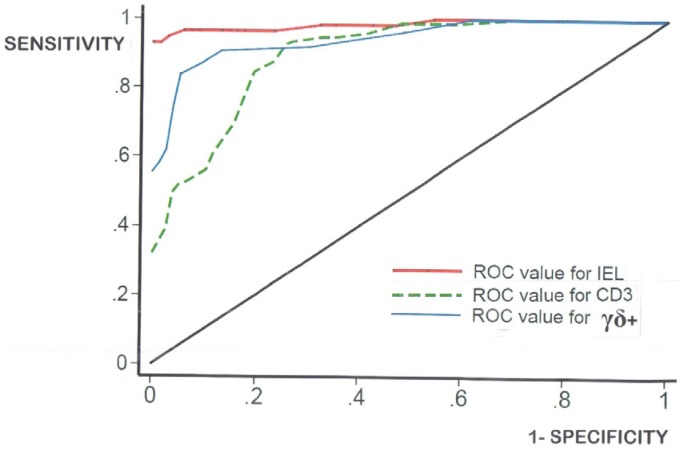
Receiver-operating characteristic (ROC)-curve analysis shows that IEL (H&E) counts per 100 enterocytes (red) are the most accurate procedure, compared with either CD3^+^(green-), or γδ^+^ (blue-) immunostained IEL. This analysis produced a cut-off of 27 IEL, with three false-negatives and six false positives. Intra-observer IEL count differences would be required to establish the degree of variation around any cut-off proposed. That important variation is often forgotten (especially if the counting has only been done by one histopathologist) and therefore rarely factored into any ‘norms’ offered. See Table 2 for further data analysis relevant to each of the three modes of IEL identified. Note, however, that the use of ROC-curve analysis considerably reduces the overlap between control and celiac biopsies (compare raw numerical distributions, Figure 2).

(c) A notional cut-off with optimal sensitivities and specificities requires calculation (Figure 4). From the data given here (Table 2), ROC-curve analysis suggests an optimal cut-off level of 27 IEL per 100 enterocytes in H&E sections, a level incurring three false-negatives and five false-positives. The results for CD3**^+^** and γδ**^+^** cells were, within this analysis, apparently less accurate, as shown comparatively in Table 2.

If there is any comfort in these results, then counting IEL in histological sections is a very useful method for differentiating control from celiac biopsies (Table 2). The difficulty arises more with mis-diagnosed (false-positive) disease controls, because other diagnostic parameters may not be available to explain a raised IEL count. 

This is diagnostically important on grounds that while repeat biopsies are often performed on celiac patients during gluten restriction, they are rarely done with disease controls. Therefore, we must be ever watchful that so-called “normal ranges” may not be a secure as some papers might suggest. This issue is critically well illustrated by one of Ferguson and Murray’s (1971) patients with “abdominal pains”. Her initial biopsy was “flat”, yielding one of the highest recorded IEL counts of 155. One year later, however, on repeat biopsy, the IEL count was then 26. The actual diagnosis and the causal reason(s) for this marked difference were never explained [28]. 

Conversely, if a celiac (nowadays) is histologically misdiagnosed (false-negative below arbitrary cut-off), other parameters (family history; DQ 2/8 haplotyping; EMA and AGA antibodies, etc.) strengthen the physician’s arm.

## 5. Objective (Computerised) Measurements of Intestinal Mucosa

One approach by Whitehead [37] used a point-counting grid producing ratios and ‘absolute’ values: however, the observer decides on which bit of mucosa the ends of the grid-lines fall, needing some degree of concentration. In Manchester, we used a test square of muscularis mucosae of 100 μm length (10^4^ μm^2^) providing an invariant reference over which we ‘rebuilt’ the mucosa in terms of villous, crypt and lamina propria volumes (as μm^3^ per 10^4^ μm^2^ of muscularis): ‘absolute’ cell counts within each space were determined independently [38], based on Weibel’s approach. 

**Figure 5:**
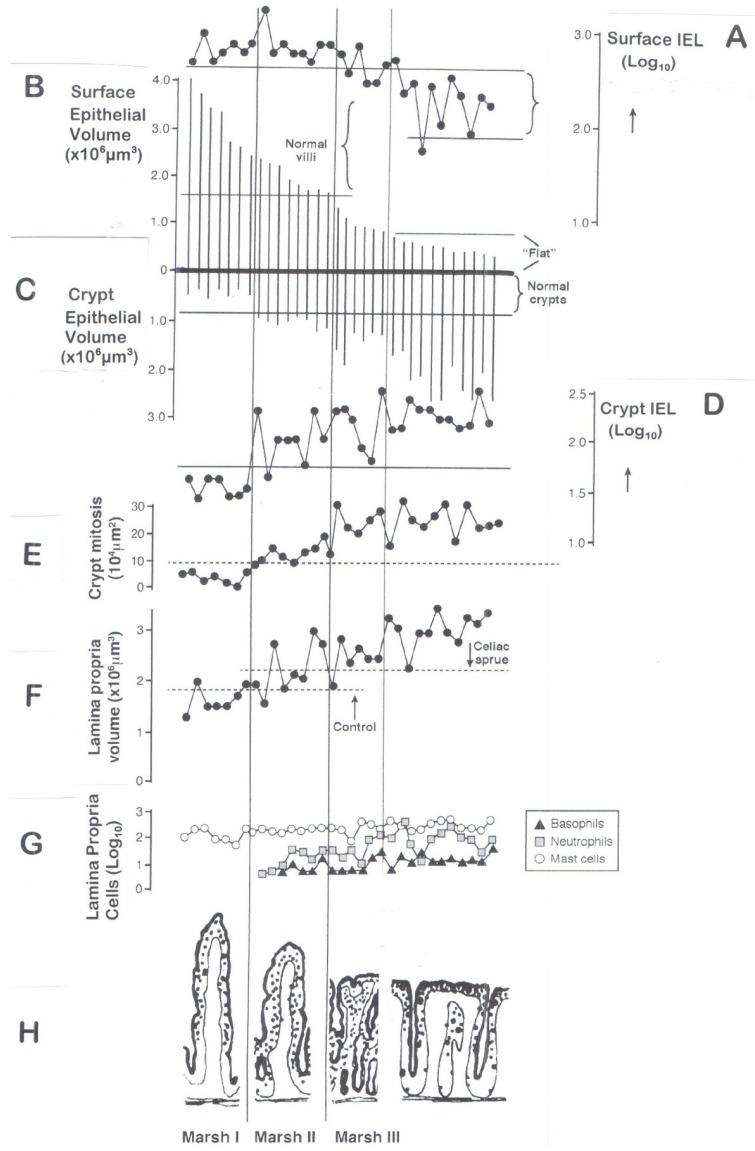
This represents mucosal metamorphosis from “normal” to “flat”, based on computerised image analysis relevant to an invariant square (10^4^ μm^2^) of muscularis mucosae. Here, a comprehensive overview provides a clear picture of the progressive morphometric**/**immunopathologic alterations observed and hardly possible by viewing a vast collection of micrographs illustrating the same changes. (**A**) This line shows the progressive reduction in absolute surface epithelial IEL populations (log_10_ transformed). With Marsh Stage III lesions, the IEL count falls *within the normal range*; (**B**) Here, volumes of surface epithelium (×10^6^ μm^3^) are shown as vertical lines, in order of flattening; (**C**) This line reveals the progressive increase in crypt epithelial volumes, which are doubled (Marsh Stage II lesions), and quadrupled (Marsh Stage III); (**D**) Changes in crypt IEL populations (log_10_) are rarely demonstrated or measured. Here we show that their number begins to change at the Marsh II Stage, progressively increasing thereafter; (**E**) Parallel with the crypt IEL rises, there is a brisk increase in crypt cell mitotic activity, which is well established at Marsh Stage II; (**F**) The lamina propria begins to increase in volume (×10^6^ μm^3^) at Stage II, indicative of marked inflammatory changes initiated within, and involving its structures; (**G**) As the lamina swells, an influx of inflammatory cells occurs (all as log_10_ counts), including basophils, mucosal mast cells, and a notably brisk rise in neutrophils; (**H**) All data are related to specific stages (Marsh I, II and III). The Marsh II lesion (despite being considered either “non-specific” or difficult to identify) enjoys a strikingly prominent role, since marked changes are already operative at this pivotal point in the sequence, indicating that the entire mucosa seems to be “active” once this stage is reached. These composite relationships have never been demonstrated in other histological studies of celiac mucosae.

This method is similar to currently employed techniques using an external scanner which takes millions of observations from an external, independent vantage point, thus creating, say, the three-dimensional structure of a jet engine or hydraulic pump, or the interior of a stately home. It is regrettable that the field of mucosal morphology has, so far, not taken advantage of such powerful computerised programmes in order to reconstruct the 3-D micro-world of the mucosa and its internal structures, especially the microvasculature. Such application to specimens undergoing regrowth during a gluten-free diet would add enormously to our understanding of the regenerative processes involved. Pseudo-colouring could also be employed to highlight structures or areas of specific interest. Our cumulative results (Figure 5) probably represent the largest assembly of data unaffected by relative measurements. When set out in this way, the data afford a panoramic view of the major structural changes taking place across the mucosa as it progressively undergoes its hypertrophic response in remodelling its surface contour. 

Some weak criticisms were raised that since the muscularis itself is caught up in the ‘mucosal celiac process’, it is invalid. But that is nonsense. Two objections arise—first, if it were thickened, which is irrelevant since we are only interested in area, and second, if it were “stretched”. We excluded the latter [39] by demonstrating identical inter-crypt distances in horizontally sectioned control and celiac biopsies.

## 6. Classification of Mucosal Remodelling: A Major Hypertrophic Process 

In overcoming these technical problems especially in circumventing inappropriate “atrophy” terminology, a novel classification [40] based on recognisable, immunopathologically phased stages (Marsh 0–III) during mucosal remodelling was proposed. Incidentally, the paper was also the first major systematic review of celiac disease, intended to divert its scientific basis away from 1950s-era thinking towards the molecular era of the 21st century. Thus, in addition to the mucosal Stage Classification, it considered possible HLA polymorphisms, relevant gliadin epitopes, and suggested a radical overhaul of lymphoma classification and treatment.

The development [40] of this classification was gradual and hesitant, depending on several contributory elements [41]: it was not an Archimedian“heuristic” of intense inspiration.

*First* came the realisation from many sporadic case reports [42,43,44,45,46,47,48] that the celiac mucosa evolves (a) over time, (b) at different rates, (c) with differing functional (clinical) outcomes, thereby (d) providing the obvious realisation that a flat mucosa is not a “given”, as was assumed from the beginning when biopsies were first observed. Histologically, the biopsies obtained in these sporadic cases were often regarded as “normal”—although some subtle changes may have been present, once the structural progression had been clarified [49].

*Second*, time**/**dose response studies on treated patients [50] showed a progression of villous infiltration, modest crypt hypertrophy, followed by flattening and finally massive increases in crypt depth. The Marsh Stage II lesion (villous infiltration with a doubling of crypt size) was seemingly identical to that described by Mowat and Ferguson [51] as a mucosal T-cell dependent phenomenon.

*Third*, came the realisation that many family relatives of known celiac patients exhibit lymphocyte-infiltrated villi with or without modest crypt hypertrophy. This observation came as we repeated the original intestinal ‘permeability’ study [52] by Tim Peters’s group in London (dealing then only with celiac patients with a flat lesion). In the repeat study [53], only celiac relatives who did *not* have gross lesional pathology were included. The realisation, for the first time, that identifiable minor changes occurred widely was, indeed, a “heureka” moment. It was very evident that lymphocytic infiltration of normal-looking villi was a frequently unrecognised but critical abnormality (except perhaps in some of the individual case reports mentioned above). But these further observations confirmed the reality of the classification, operative now for 25 years, which incorporates the major phases in the immunohistological progression to a flat lesion (Figure 5). 

Based on that classification, we are now in a position to evaluate the structural remodelling of the mucosa.

### 6.1. The Surface Epithelium

We use Figure 5 for guidance. The top line, A, in this diagram represents the IEL population, expressed logarithmically (with absolute counts) and its progressive reduction towards the control range with mucosal flattening. In comparison, the progressive reductions in villous surface volumes are expressed as vertical lines along the second horizontal strand (B). The mean volume for an infiltrated mucosa is 2.6 (1.5–3.6) × 10^6^ μm^3^, compared with 0.4 (0.2–0.6) × 10^6^ μm^3^ for flat lesions: that is, a five-sixths volume reduction. 

But these data can be interrogated further, in respect of IEL populations, because we also measured [54] individual cell volumes. These allowed us to calculate the number of enterocytes within each specified volume of epithelium, from which absolute ratios of IEL per 100 enterocytes could be determined, as follows. 

Average cell volumes were 780 μm^3^ (~800) for control enterocytes compared with 600 μm^3^ for flat mucosae, although we do not know why Stage III enterocytes suffer 25% volume reductions. From those measurements, the absolute population of enterocytes in surface epithelium is ~3000 compared with 600 enterocytes for flat specimens. Flattening thus incurs a ~80% loss of surface enterocytes. Therefore, using data (line A, Figure 5), celiac disease specimens contain 190 (150–240) IEL, representing a less marked reduction of ~50% compared with enterocyte losses (Table 3). That is why, relatively, a flat mucosa *appears* to be infiltrated by lymphocytes: in fact, that is clearly not the case.

Second, use of ‘absolute’ data permits determining ratios of IEL per 100 enterocytes (Figure 3). For diseasecontrols the values were 12 (10–16), and 32 (27–37) for flat lesions. However, when the same specimens were counted according to Ferguson (using 1 μm toluidine blue-stained Epon sections viewed under oil immersion optics), the values were *doubled* over the absolute counts: 24 (11–53) for disease controls, and 61 (31–122) for celiacs. 

**Figure 6:**
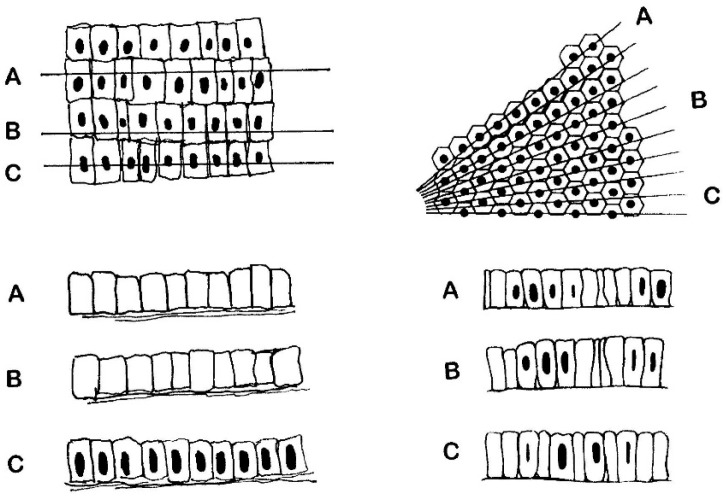
The **left-hand panel** indicates that enterocytes do not lie in an orthogonally arranged grid pattern on the basement membrane (plan view, upper diagram). Therefore, “counts” of enterocytes (or more importantly their nuclei) cannot be accomplished with the ease often assumed in the Methods sections of many publications. This model obviously predicts the possibility of observing large tracts of enterocytes without nuclei (as in the imagined sections at (**A**,**B**)), an event never encountered in histopathological practice. Only occasionally would a palisade that included a run of every adjacent enterocyte, and their contained nuclei, be observable (**C**). Therefore, this model is wrong. The alternative (**right-hand panel**) is closer to reality, comprising an idealised epithelium, scaled to data obtained by transmission**/**scanning EM studies. The lines in the upper (plan) diagram reflect random sectioning planes through this epithelium. But, it should be carefully noted that, on average, only ~50% nuclear profile discs appear in any section, as represented imaginatively in A,B,C below. Thus, the high numbers of “lost” enterocyte nuclei now becomes apparent. However, since IEL counts are made relative to the simultaneously changing world of enterocyte (nuclei) populations, values are spuriously increased twofold. The basic flaw is discussed elsewhere (reference [55]: and see Figure 5).

This difference is greatly significant, and rests on the failure of the Ferguson technique to identify every epithelial cell thought to have been counted. The deficit results from the fact that only enterocyte nuclei are counted [55] and not individual epithelial cells, which cannot be sequentially identified during counting. The difference between nuclei counted (rather than individual enterocytes) is of the order of a 50% reduction, resulting in the spuriously doubled IEL count. The inherent problems are illustrated (Figure 6). The basic problem is the attempted matching of one moving variable against another: a no-win situation.

The alternative (right-hand panel) is closer to reality, comprising an idealised epithelium scaled to data obtained by transmission/scanning EM studies. The lines in the upper (plan) diagram reflect random sectioning planes through this epithelium. It should be carefully noted that, on average, only ~50% nuclear profile discs appear in any section, as represented imaginatively in A,B,C below. Thus, the high numbers of “lost” enterocyte nuclei now becomes apparent. However, since IEL counts are made relative to the simultaneously changing world of enterocyte (nuclei) populations, values are spuriously increased twofold. The basic flaw is discussed elsewhere (reference [56]: and see Figure 5).

From that (Ferguson) position, nevertheless, it is usually asserted that IEL are increased within flat mucosae, but that needs qualifying. The computerised data reveal an absolute six-fold reduction in enterocytes for flat mucosae, whereas the IEL population is only reduced two-fold. Therefore, *relatively*, the IEL density obviously remains high, as inferred correctly [15] by Guix and Whitehead. Further proof is afforded by other calculations made possible by our approach, since a single IEL is associated with 9 (7–11) enterocytes in control mucosae, but only 3 (2–4) enterocytes in flat celiac mucosae, emphasising the markedly increased “concentration” of IELs in flat biopsies, largely exaggerated by the precipitous loss (80%) of surface enterocytes. On those grounds, how would we answer the critical question ‘Is the flat mucosa actually infiltrated at Marsh Stage III, and by what extent’?

It has also been shown [56] that IEL in flat mucosae are considerably larger than those in control mucosal specimens but it is unlikely that these are gluten-induced ‘blasts,’ as they would presumably be of similar calibre in the early infiltrated Stage I and II lesions. The lymphocytes in these early lesions, however, are small and non-mitotic. It is possible that, resulting from widespread shedding of the surface epithelium, some attempt at repairing a depleted IEL population from within the epithelium is operational. In support of that idea, we have to take into account [57,58] the raised mitotic activity of IEL in flat (Marsh III) mucosae. But that is another problem remaining to be resolved, as well as the immunophenotype of lymphocytes involved. We are totally ignorant of those details.

### 6.2. The Crypts

In comparison with the great interest in surface epithelium, the crypts have always played the “Cinderella” role, as the forgotten companion. In earlier studies [59] from Trier’s lab in Boston, the use of mucosal explants revealed the rapidly accelerated flow of cells upwards towards the surface, complementing previous washout studies which likewise suggested a massive loss of enterocytes in untreated patients. That was followed by Nicholas Wright’s elegant investigations which showed [60] that (i) the growth fraction in the crypts is enlarged; (ii) the actual duration of crypt cell mitosis is shortened (from the normal rate of 1 h to approximately 40 min); and (iii) the inter-mitotic interval is reduced, so that successive mitoses are speeded up. These observations revealed the degree to which the hypertrophic crypt response is geared up for the assumed losses of surface enterocytes.

In Figure 5C, it is evident that the crypts are small and non-infiltrated in mucosae where villi are subject to infiltration. But things change markedly as flattening proceeds (Marsh Stage II) with a doubling of crypt volumes and increased lymphocytic infiltration (Figure 5D) accompanied by the first evidence of increased crypt cell mitotic activity (Figure 5E). These changes are highly reminiscent of the enlarged crypts together with normal, infiltrated villi [61] in mild graft-versus-host reactions where recipient and donor tissue were of identical genetic histocompatibility backgrounds: a phenomenon termed the ‘innocent bystander effect’ [62] in the intestine by Elson. 

It is thus evident that the mucosal (Marsh) Stage II development reveals important outcomes, since additional mechanisms are now clearly in place which progress lesion pathology towards its final state. It is questionable whether the progressive hypertrophy of the crypts to almost four-fold (once flattening has been achieved) is still a continuing T-cell-mediated effect, or whether loss of surface cells still has to be accommodated by a massively increased crypt cell production rate, and migratory profile. Neither do we know why the initial infiltration of crypts is delayed, why the later increased lymphocyte infiltration does not impair their vast hypertrophic crypt responses even though mucosal surface contours are reduced, or why crypt IEL are significantly enlarged over control mucosae [63], although of similar size distribution to surface IEL.

### 6.3. The Lamina Propria

Further evidence for this proposal is seen in the lamina propria (Figure 5F) which has begun to swell at Stage (Marsh) II, accompanied by a brisk influx of neutrophils, always indicative of mucosal inflammation and a rise in basophils and mucosal mast cells (Figure 5G), many seemingly degranulated [64]. That reflects the two-fold swelling of lamina propria partly due to local vasodilatation of the microvasculature whose vessels are swollen, with enlarged endothelial nuclei, thickened basal laminae, and cells such as eosinophils and basophils emigrating across their walls into the surrounding tissues [65]: fibrinogen staining provides a rough indicator of the extravasated vascular fluids.

Current celiac research seems to have lost sight of the influence of mucosal mast cells, their T-cell dependency [66,67,68] and contributory roles in the evolutionary genesis of the celiac lesion, especially within the subepithelial zone, and contributors to the local T-cell-mediated hypersensitivity reaction to gluten. Computerised morphometry showed that mucosal mast cell populations are increased 2.5 times, eosinophils 4.5 times, and basophils 20-fold over control values, and all gluten dependent. The influx of eosinophils and basophils through the microvasculature suggests a bone marrow origin. Mucosal mast cells were never seen in the vascular compartment, so are presumably differentiated locally, or from incoming precursors not distinguishable histologically.

## 7. Interpreting the Marsh Classification 

The changes noted in this diagram (Figure 5) as the mucosa progresses from villous infiltration to flattening is illustrated (Figure 5H) by appropriate diagrams (Marsh Stages I through III). It is to this classification of the mucosal changes that we now pass.

### 7.1. So-Called “Non-Specificity” of the Marsh I and II Lesions

Many have dismissed early Marsh I and II lesion as ‘non-specific’ [69,70]. On the other hand, there are those who have understood that Marsh I/II lesions should be investigated prospectively [31], thus to exclude true glutensensitivity: as these authors summarise—*’a raised IEL count with normal villous architecture is of sufficient clinical importance to be highlighted in routine duodenal biopsy reports’.*

To clarify this position for histopathologists, a series of differential diagnoses has been set out by the Bucharest Consensus [71], under the terminological umbrella of “microscopic enteritis”. It is to be hoped that these widelyagreed guidelines will be recognised and employed. And within a family setting and DQ 2/8 haplotypes, the possibility of celiac disease remains a high probability. Individuals with these mucosal changes should be closely followed up, or even treated [72], particularly if they have disabling symptoms associated with malabsorption of important nutrients. Despite a lesser mucosal involvement there is often considerable abdominal symptomatology and pain, osteoporosis and iron deficiency anemia, features surely necessitating a gluten-free diet—even only if a defined, agreed, short-term trial to monitor clinical response and reversal of malabsorptive defects is undertaken. Given the growing literature, it is now *unacceptable to refuse a diet on the grounds that the mucosa is not* flat. By now, it should be widely recognised that there is neither a specific, nor certainly a uniquely related diagnostic mucosal change.

### 7.2. Irrelevance of the Marsh III Sub-Classification

The division [73] of the Marsh III lesion into three subdivisions (a, b, c), as a “guide to histopathologists” has been widely, but surprisingly uncritically, employed. This proposed analytical system is a failure because of the following flaws:

(a) *Absence of appropriate criteria*: these subdivisions were never precisely defined morphologically as verification of the proposed subdivisions. It is interesting to envisage how (and why) so many histologists thought they were identifying real structures. Even the micrographs illustrated in a later publication [74] written by histopathologists, for the help of other histologists, failed to correspond to the originals, again demonstrative of the subjective nature of the whole scheme.
  Oberhuber’s approach has now been further degraded by additional studies:
(b) *morphological—*which highlight the misinterpretations of sectioned mosaic plateaus as supposedly representing ‘blunted’, ‘degenerate’ ‘villi’ [75];
(c) *immunohistochemical*—demonstrating that varied sub-immunophenotype IEL are equally represented in each subdivision, when their density should have increased with the worsening histological picture alleged to represent each successive stage: a, b, c [76];
(d) *mathematical*—the regression equations employed by Charlesworth and colleagues failed to identify the a,b,c subgrades as valid entities for improved pathological recognition [77];
(e) *clinical*—there appear to be no published accounts in which a gastroenterologist necessarily had to rely, ultimately and crucially, on the pathologist’s *sub-classification* of the relevant mucosal biopsy in order to facilitate diagnosis, treatment, or offer a prognosis for the patients concerned;
(f) *generalised usage*—finally, given the failure of this attempted reclassification, it seems to follow that more recently revised classifications of Marsh were based, however, on these sub-divisions, offering no further decisive clarity. In fact, they could be said to increase complexity and interpretational difficulties. For example, from a review of relevant papers published over the last decade, it is abundantly clear that these recent contenders for the job have not surfaced either as being more useful, more acceptable, or more easily employed. The original classification is as simple as could be.

### 7.3. The “Normal” Mucosa

Finally, we come to the interpretation of the ‘normal’ (Marsh Stage 0) mucosa. One problem concerns origins of specimens—from referred, symptomatic patients or apparently healthy individuals. There *are* differences—but which nowadays are rarely considered or explored (see last paragraph: Immunological Function of Mucosa, above). Second, ‘normality’ is no longer defined, although from early times, villi were seen as long, pencil-shaped structures 350–600 μm in height [9].

Overriding those relevant considerations, however, is the recognition that ‘normal’ mucosae, viewed histologically, may be consistent with gluten sensitivity, harbouring abnormalities requiring additional but difficult technologies for detection, including immunofluorescence of anti-TG antibodies on epithelial and microvascular basement membranes [78]; transmission EM detection of necrotic enterocytes [79,80], or assays of fatty acid binding protein as presumptive indicator of cell death [72]. 

### 7.4. Failures in Understanding the Marked Hypertrophic Remodelling Response

The problems arising from the sub-classification of the Marsh III lesion stem from the continuing belief that mucosal flattening strips every single villus down to the crypt-villus border, supposedly considered the end-stage of a progressive, atrophic process. There is no morphologic evidence for that presumption. The changes that involve most of the mucosa (excepting epithelium) represent the effects of considerable remodelling, embodying a vast hypertrophic response in terms of the upward growth and enlargement of the inter-villous ridges, and their amalgamation with partially reduced villi to create irregular mosaic plateaus over the mucosal surface, with height elevations of ~200 μm.

The hypertrophic response is further exemplified by the vast increase in the size of the crypts, their infiltration by a population of large IEL, and the increased dynamic of the ascending enterocyte column in its movement towards the surface. The lamina also swells to twice its volume due to extravasation of plasma fluid through the inflamed capillaries, and great increases in the bulk of infiltrating cells. This is a complex epithelial-mesenchymal response indeed, and a markedly dynamic hypertrophic response to gluten. 

It seems that this end-phase of mucosal flattening is not generally wellunderstood. As a result, random sections through the mosaic plateaus create a variety of appearances which histologically are invariably taken to represent stunted or branched villi. Surface microscopy, however, does not reveal the presence of any villi, so these structures seen two-dimensionally merely reflect the many possibilities on offer when a mosaic plateau is observed in any random section.

This state-of-affairs is scarcely helped by current, expert guidelines [81,82] whose authors collectively provide no incisive practical outcomes from the literature. The guidelines signally fail to engender the vital cooperative understandings required between pathologist and clinician regarding mucosal interpretation. In fact, these guidelines do not confidently explore the full spectrum of mucosal abnormalities of gluten-induced mucosal change, being more at ease with “atrophy” and the flat lesion. As a result (a) they tend to dismiss all other preliminary phase transitions as “non-specific”; (b) rely on traditional definitional criteria—that is, ”atrophy”—resulting in a flat mucosa and (c) are hesitant to recommend a gluten-free diet without that latter criterion, despite a very large literature to the contrary [31,32,69,70,71,72,80,83].

There is a pressing need to reconstruct biopsies with computerised programmes, using either the systems of indices and matrices employed in computer-assisted design, or by employing 3-D printing. Such approaches would further expand our understandings of the mucosa, and its internal changes, especially where the remodelled microvasculature is concerned. If we knew more about the effects of gluten on the small vessels and how they influence the hypertrophic responses throughout the mucosa, we might be in a more enviable position to understand how these changes come about—both in their association with flattening as much as with regrowth. There is much to be re-remembered from the past, organised from the present, and planned for the future [15].

## 8. Afterword

Ptolemy may have been a little disgruntled when his geocentric theory was overtaken by the more ambitious heliocentric-based Copernican view of the universe. Yet it hardly seems time to declare that celiac disease has become so universalised that the intestinal tract has been side-stepped and no longer plays a central role in furthering insights into the disease: that seems to us to be a misleading—if not premature—conclusion. 

From all this it should be clearly understood that:

1. there are no (immuno)histologically unique diagnostic features for celiac disease that “absolutely” distinguish it from other mucosal enteropathies or more importantly, disease-control biopsies;
2. the spectre of the “normal” mucosa, but which is consistent with true gluten sensitivity, remains a difficult problem to deal with, including its redefinition;
3. there is considerable overlap between the populations of celiac intraepithelial lymphocyte (IEL) and controls (Figure 2)—regardless of the identifying technique used;
4. IEL populations do not comprise two separate populations (bimodal), but represent graded biological outcomes (to luminal antigens), analogous to height, weight, blood pressure or acid secretion (Figure 3);
5. additionally detailed studies of the dose-response characteristics of the CD3**^−^** innate pool of IEL, and their CD127**^+^** and CD127**^−^** components may bring new insights to diagnosis and mucosal interpretation;
6. ROC curve analysis (Figure 4 and Table 2) provides usable answers which overcome the immense numerical overlapping between IEL populations, including CD3^+^ and γδ^+^ cells, and removes to a great extent the inherent uncertainty, engendered with numerical counts, as to where to draw the cut-off;
7. log-transformation of the skewed celiac data does not produce means which materially differ from the numerical means (data not shown). Together, these results confirm that histopathologists do not need to log-transform their numerical counts, and that IEL counts in routine hematoxylin and eosin (H&E)sections can now be seen as a very easy and resourceful way of defining one’s cut-off, provided receiver-operating characteristic (ROC) curve analysis is additionally carried out;
8. there is a vast cavern between high-level research still needed in continued interrogations of the mucosal response to gluten ingestion, and the somewhat more unsophisticated approaches deployable at histopathological level during routine diagnostic service work.

Notwithstanding those difficulties, the tracing of the historical development of our understandings of the structure and functioning of the small intestinal mucosa is a truly fascinating story. Our own view is that the mucosa still occupies a very central role in diagnosis and, together with related research, into its response to gluten peptides.

There is a long list of historic figures who have welded the story of the intestinal mucosa into one which still causes dissent, re-evaluation, and the pull of additional research initiatives. That is the true nature of investigative science, and there will surely be more advances to clarify, and to strengthen our grasp on this important field of gluten-induced hypersensitivity reactions within the intestinal mucosa. 

## Figures and Tables

**Table 1:** Summary of papers on intraepithelial lymphocytes (IEL) counts.

Paper	Methods	Number of Biopsies	Upper Range	Comments
Ferguson and Murray, 1971 [28]	H&E staining IEL/100 enterocytes	40	40	Used controls, celiac and autoimmune conditions. Incorrect about normally distributed IEL. Highest IEL count recorded, of 155
	7 μm sections			
Batman et al., 1989 [29]	H&E staining 5 μm sections	8	33	Study of HIV enteropathy
Hayat et al., 2002 [30]	H&E staining	20	25	Counts made on uninterrupted length of epithelium >500 epithelial cells: Controls defined only by a “normal” sugar permeability
	4 μm sections			
Mahadeva et al., 2002 [31]	H&E staining	??	22	Major interest in normal villi with IEL infiltrate
	3 μm sections			Really difficult to infer group numbers here
Kakar et al., 2003 [32]	H&E staining	12	39	Interest in normal villi with IEL infiltrates
Veress et al., 2004 [33]	H&E staining	64	20	3 μm H&E sections:
	CD3^+^ counts		5–9	If IEL to EC ratio >5:1, do CD3 count
Biagi et al., 2004 [34]	H&E staining	17	45	Major interest in villous tip counts
Nasseri-Moghaddam et al., 2008 [35]	H&E staining	46	46	Establishing normal criteria by histology and immuno-cytology
	CD45^+^ counts		47	
Siriweera et al., 2015 [36]	H&E staining	75	8	Retrospective study on 38 control specimens and 37 celiacs. Inexplicably small upper ranges for both groups

**Table 2:** Summary of ROC-curve Analyses.

Lymphocyte Subtype	H&E Stained	CD3^+^	γδ^+^
AUC	0.985	0.891	0.943
OPTIMALCUT-OFF	27	40	6
FALSE-POSITIVE	5	21	10
FALSE-NEGATIVE	3	11	15

**Table 3:** Numerical values for intestinal mucosa (computerisedimage analysis).

	Disease Controls	Celiac Disease
Surface Epithelium		
Volume (×10^6^ μm^3^)	2.3 (1.5–3.6)	0.4 (0.2–0.6)
Cell Height (μm)	37 (30–43)	33 (27–33)
Cell Width (μm)	5.1 (4.1–6.2)	4.7 (3.8–5.8)
Cell Volume (μm^3^)	800 (500–1250)	600 (390–920)
No. Enterocytes/Volume	3000 (1935–4435)	600 (320–1100)
No. IEL/Volume	350 (275–450)	190 (150–240)
IEL/100 enterocytes		
(‘Absolute’ by Image Analysis)	12 (10–16)	32 (27–37)
(Ferguson, per 100 cells)	24 (11–53)	61 (31–122)
Enterocytes per Lymphocyte	8 (7–11)	3 (2–4)
CRYPTS		
Volume (×10^6^ μm^3^)	0.5–0.6	1.7
IEL (‘Absolute’/volume)	30 (12–48)	173 (121–225)
LAMINA PROPRIA		
Volume (10^6^ μm^3^)	1.4 (1.12–1.6)	3.1 (2.8–3.5)
Cells/Volume		
(‘Absolute’)		
Mast Cells	14 (10–20)	38 (22–54)
Eosinophils	18 (16–20)	62 (50–74)
Basophils		0.7 (0.48–1.12)
Neutrophils		45 (25–65)
